# Association of myosteatosis with various body composition abnormalities and longer length of hospitalization in patients with decompensated cirrhosis

**DOI:** 10.3389/fnut.2022.921181

**Published:** 2022-09-15

**Authors:** Xiaoyu Wang, Mingyu Sun, Yifan Li, Gaoyue Guo, Wanting Yang, Lihong Mao, Zihan Yu, Yangyang Hui, Xiaofei Fan, Binxin Cui, Kui Jiang, Chao Sun

**Affiliations:** ^1^Department of Gastroenterology and Hepatology, Tianjin Medical University General Hospital, Tianjin, China; ^2^Tianjin Institute of Digestive Disease, Tianjin Medical University General Hospital, Tianjin, China; ^3^Department of Gastroenterology, Tianjin Medical University General Hospital Airport Hospital, Tianjin, China

**Keywords:** myosteatosis, liver cirrhosis, visceral adipose tissue, adipose tissue depot, risk factor, sarcopenia

## Abstract

**Background:**

Myosteatosis is linked to dismal outcomes in the context of cirrhosis. However, the association of myosteatosis with various body composition abnormalities remains enigmatic. We aimed to clarify the determinants of myosteatosis and its relationship with other body composition profiles and length of hospitalization (LOH).

**Methods:**

We retrospectively analyzed the data of 473 consecutive patients with cirrhosis hospitalized for decompensation. Computed tomography-based segmentation of the cross-sectional area at the third lumbar vertebra was used to evaluate body composition abnormalities. The categories of myosteatosis were built according to our previously outcome-based cutoffs for each gender.

**Results:**

Totally, 83 patients (17.55%) were stratified as myosteatosis, of whom 85.54% had concomitant high visceral adiposity indicative of increased visceral adipose tissue index (VATI). The prevalence of sarcopenia showed no significant difference between the groups with and without myosteatosis. Multivariate analysis showed that advanced age [odds ratio (*OR*) = 1.097, *p* < 0.001], higher visceral to subcutaneous ratio of adipose tissue area (VSR; *OR* = 1.574, *p* = 0.032), and higher VATI (*OR* = 1.026, *p* < 0.001) are independently associated with myosteatosis. Correlation analyses revealed a positive relationship between intramuscular adipose tissue content (IMAC) and VATI (ρ = 0.48, *p* < 0.001), subcutaneous adipose tissue index (SATI) (ρ = 0.36, *p* < 0.001), and age (ρ = 0.36, *p* < 0.001). None of the skeletal muscle or adipose tissue indicators were significantly related to longer LOH.

**Conclusion:**

Higher VSR, higher VATI, and advanced age are associated with myosteatosis among patients with cirrhosis at the decompensation phase. It is tempting to target divergent adipose tissue depots aimed at timely intervention/prevention of myosteatosis.

## Graphical Abstract

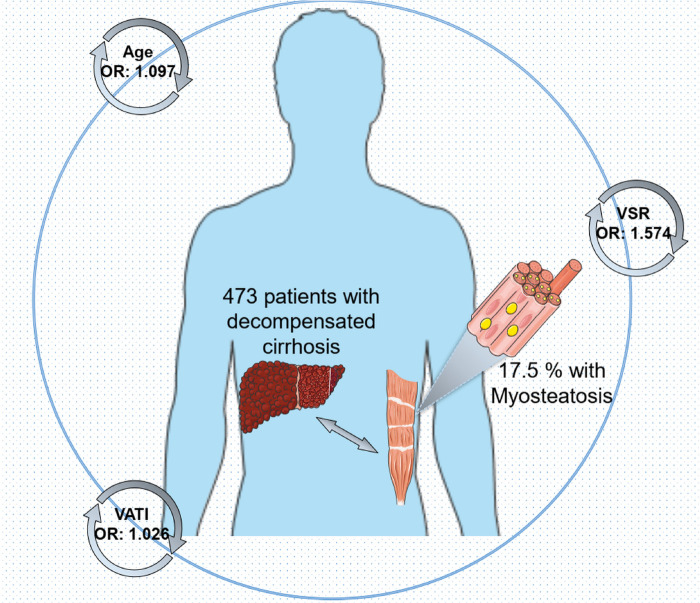

## Introduction

Over the past decade, an increasing number of studies have found that the status of the skeletal muscle compartment affects the prognosis of various end-stage liver diseases. Previous reports demonstrated the association of reduced skeletal muscle mass (abnormal muscle quantity), also known as sarcopenia, with adverse outcomes in the context of cirrhosis (1, 2). On the other hand, ectopic adipose tissue disposition in the skeletal muscle called “myosteatosis” (abnormal muscle quality) has recently been independently linked to an increased risk of inferior outcomes, worsening physical status, and debilitating conditions among cirrhotics (3–5). Evaluation of muscle quality is gaining interest in accordance with the guidelines proposed by the European Working Group on Sarcopenia in Older People (EWGSOP), highlighting the clinical significance of muscle quality equal to (or even superior to) muscle quantity (6). A recent investigation by our group has clarified not only a considerable prevalence of myosteatosis but a close relationship with dire outcomes in patients with cirrhosis hospitalized for decompensation (7).

In addition, unlike sarcopenia, factors in relation to the presence of myosteatosis are still open questions in the setting of hepatic cirrhosis, and scattered reports have concentrated on this issue. Looking into the existing literature, we found that Tachi and colleagues demonstrate that older age, female gender, presence of sarcopenia, and higher levels of visceral to subcutaneous ratio (VSR) of the adipose tissue area are independently associated with myosteatosis (8). Furthermore, the authors addressed myosteatosis is a remarkable predictor of sarcopenia and this pathological entity is present in 93% of patients with sarcopenia. However, the aforesaid study only enrolled a fraction of patients with cirrhosis with well-preserved hepatic function at Child-Turcotte-Pugh classification A (CTP-A, 26.0%). In contrast, another research has implicated that myosteatosis can be influenced by various clinical factors and does not accompany sarcopenia (9). It has also been proven that there is a lack of interaction between sarcopenia and myosteatosis in a cohort of 64 patients with cirrhosis (10). Notably, myosteatosis is more than the result of fat tissue saturation or aging but also a consequence of combined mechanisms, such as muscle damage, mitochondrial dysfunction, chronic inflammation, and hormone dysregulation (11). Taken together, we aimed to investigate the interaction between myosteatosis and a wide range of body composition abnormalities, demographic characteristics, and biochemical indices, which may facilitate the development of potentially effective intervention in the context of decompensated cirrhosis.

## Materials and methods

### Study population

All consecutive patients hospitalized for decompensated events between 2017 and 2021 in the Department of Gastroenterology and Hepatology, Tianjin Medical University General Hospital (TJMUGH), were considered for inclusion in the present retrospective analyses. Patients without computed tomography (CT) imaging 3 months prior to index hospital admission or during hospitalization were not eligible for the study. Participants with acute-on-chronic liver failure, primary hepatic tumors/extrahepatic cancer, and liver transplantation were also excluded. This study corresponded to the principles of the Declaration of Helsinki and was approved by the ethics committee of TJMUGH (IRB2021-YX-136-01). Informed consent was obtained from all patients.

### Image analyses

CT images and their segmentation of the cross-sectional area were retrieved. In brief, image data of the most recently performed CT scan were analyzed by one of the investigators (G.Y.G. or W.T.Y.) who was blinded to outcomes and the remaining clinical features of the patients. A single cross-sectional image at the third lumbar vertebra (L3) has been analyzed by using a program on the basis of the MATLAB version R2010a (Mathworks Inc, Natick, MA, United States) as described before (12). The definitions and well-built cutoffs applied in the segmentation analyses are illustrated in Table 1. It is pivotal for correcting the gender-associated differences in muscle and adipose tissue volume and radiodensity to generate gender-specific (male-female) cutoffs. These cohort-specific cutoffs have been defined in terms of optimal reference values pertaining to patient mortality as previously depicted in our study (7). Although a standardized diagnostic modality for myosteatosis has not been constructed, we evaluated the muscle quality by using intramuscular adipose tissue content (IMAC) at the L3 level. Actually, this radiodensity-derived novel parameter has been widely implemented in multiple Japanese studies, which holds the promise to eliminate the variation between individual CT scans and patients, resulting in improved recognition of clinically significant alterations (13, 14). Increased IMAC implicates a higher amount of adipose deposition in the muscle tissue, thus a lower muscle quality. In addition, a spectrum of adipose tissue depots and muscle mass was calculated and normalized to stature (m^2^) including subcutaneous adipose tissue index (SATI), visceral adipose tissue index (VATI), total adipose tissue index (TATI), and skeletal muscle index (SMI). Notably, VSR is referred to as abdominal abnormal adiposity due to imbalanced adipose depots (visceral-subcutaneous). In addition, skeletal muscle radiodensity (SMR) was assessed and recorded as the mean value for the muscle cross-sectional areas entirely.

**TABLE 1:** Body composition abnormalities and gender-specific cutoffs.

Body composition abnormalities	Area and definition	Interpretation	Patient cutoffs	
			Female	Male
IMAC	L3 region of the interest (ROI) of the multifidus muscle/(ROI) of subcutaneous adipose	Increased values imply more low attenuation myosteatotic muscle, thus lower muscle quality	>−0.37	>−0.44
SMI (cm^2^/m^2^)	L3 whole skeletal muscle area/squared height	Decreased values imply wasting muscle mass, thus lower muscle quantity	<32.46	<46.96
VATI (cm^2^/m^2^)	L3 whole visceral adipose tissue area/squared height	Increased values imply excessive accumulation of adipose tissue in the visceral depot, thus high visceral adiposity	>44.02	>28.42
SATI (cm^2^/m^2^)	L3 whole subcutaneous adipose tissue area/squared height	Decreased values imply insufficient storage of adipose tissue in the subcutaneous depot, thus low subcutaneous adiposity	<26.75	<29.1
VSR	Visceral to subcutaneous ratio of adipose tissue area as described above	Higher ratio implies unbalanced distribution of visceral against subcutaneous adipose depot, thus abnormal adiposity	>1.29	>1.47

IMAC, intramuscular adipose tissue content; SMI, skeletal muscle index; VATI, visceral adipose tissue index; SATI, subcutaneous adipose tissue index; VSR, visceral to subcutaneous ratio of adipose tissue area. We used the following attenuation cutoffs to discriminate between various tissue components on cross-sectional CT scans according to literature definitions: Skeletal muscle: −29 to 150 HU, visceral adipose tissue: −150 to −50 HU, and subcutaneous adipose tissue: −190 to −30 HU. The gender-specific cutoffs are on the basis of patient mortality in terms of our prior investigation.

### Clinical and laboratory information collection

We collected the clinical and laboratory information from our prospective institutional database and analyzed it in a retrospective manner. Age, gender, body mass index (BMI), indication with respect to decompensated insults (gastroesophageal varices, ascites, hepatic encephalopathy, and infection), conventional scoring systems [CTP and model for end-stage liver disease (MELD) score], platelet, serum creatinine, biomarker of inflammatory action [neutrophil-to-lymphocyte ratio (NLR), lymphocyte-to-monocyte ratio (LMR), and platelet-to-lymphocyte ratio (PLR)], hepatic function tests [alanine aminotransferase (ALT), and aspartate aminotransferase (AST)], albumin, prothrombin-international normalized ratio (PT-INR), and sodium were extracted from electronic medical records. The length of hospitalization (LOH) was calculated and dichotomized according to the median value designated as the in-hospital outcome. Correspondingly, we regarded LOH exceeding the median duration as “longer LOH.”

### Statistical analyses

The Shapiro–Wilk test was used to test the normal distribution for continuous data. Continuous data were presented as median [interquartile range (IQR)], while categorical data were presented as absolute and relative frequencies appropriately. The Student’s *t*-test and the Mann–Whitney *U*-test were used for the statistical comparison of continuous data. In the case of comparing categorical data, the chi-square test or Fisher’s exact test was used. To identify determinants of myosteatosis, uni- and multivariate binary logistic regression analyses were performed. For appropriate interpretation in clinical practice, we established two regression models by examining body composition profiles as both continuous and dichotomized variables (SMI/sarcopenia, VSR/abnormal adiposity, etc.) The odds ratio (*OR*) was calculated, as well as a corresponding 95% confidence interval (*CI*). Spearman’s rank correlation analyses (ρ) were adopted to further analyze the correlations between the continuous data. We considered the level of statistical significance as *p* < 0.05 and all statistical analyses were carried out by using SPSS 23.0 (IBM, New York, NY, United States) or R software 3.3.2.^1^

## Results

### Study cohort and characteristics

Of the 473 consecutive patients with cirrhosis who were hospitalized for decompensated events within the given investigation period, 238 patients (50.32%) were women with a median age of 66 (IQR: 55, 69) years. The most common indications for hospitalization were ascites (43.13%), gastroesophageal variceal bleeding (42.28%), and infection (13.74%). For scoring systems pertaining to disease severity, the study population had median MELD scores of 10 (IQR: 8, 13) points. The numbers of enrolled participants at CTP-A/B/C were 131, 288, and 54, respectively. The major etiology of cirrhosis was attributed to hepatitis B or hepatitis C viral infection (28.96%), which was followed by metabolic dysfunction-associated fatty liver disease and autoimmune liver disease (27.27%). Detailed patient characteristics are shown in Table 2.

**TABLE 2:** Baseline characteristics of study population in different groups stratified by myosteatosis.

	Total	Myosteatosis		*P*
	(*N* = 473)	No (*N* = 390)	Yes (*N* = 83)	
Age (years)	63(55,69)	61(53,68)	69(63,78)	<0.001
Sex, n (%)				0.277
Male	235 (49.68)	189 (48.46)	46 (55.42)	
Female	238 (50.32)	201 (51.54)	37 (44.58)	
CTP, n (%)				0.038
A	131 (27.69)	115 (29.49)	16 (19.28)	
B	288 (60.89)	236 (60.51)	52 (62.65)	
C	54 (11.42)	39 (10)	15 (18.07)	
MELD score	10(8,13)	10(8,13)	10(8,13)	0.701
Etiology, n (%)				0.436
HBV/HCV	137 (28.96)	117 (30)	20 (24.10)	
Alcohol	100 (21.14)	85 (21.79)	15 (18.07)	
MAFLD/AILD	129 (27.27)	104 (26.67)	25 (30.12)	
Cryptogenic/Others	107 (22.63)	84 (21.54)	23 (27.71)	
**Complications, n (%)**				
Ascites	204 (43.13)	165 (42.31)	39 (46.99)	0.465
Hepatic encephalopathy	54 (11.42)	41 (10.51)	13 (15.66)	0.185
Gastroesophageal varices	305 (64.48)	262 (67.18)	43 (51.81)	0.011
Infection	65 (13.74)	53 (13.59)	12 (14.46)	0.861
DM				0.152
No	391 (82.66)	327 (83.85)	64 (77.11)	
Yes	82 (17.34)	63 (16.15)	19 (22.89)	
LOH (days)	13(10,17)	13(10,17)	13(9,16)	0.612
Platelet (*10^9^/L)	76(54,113.50)	76(54,114.30)	76(54,112)	0.830
Sodium (mmol/L)	140(137,142)	140(137,142.50)	140(137,142)	0.472
Albumin (g/L)	30(26,34)	30(26,34)	29(25,32)	0.070
Total bilirubin (μmol/L)	21.60(14.70,37)	21.30(14.45,37)	23.10(16.80,37.40)	0.358
ALT (U/L)	21(15,36)	21(14,36)	23(16,34)	0.458
AST (U/L)	31(23,51)	30(22,49.75)	35(26,55)	0.114
Creatinine (μmol/L)	61(51,77)	61(51,77)	62(50,80)	0.809
PT-INR	1.26(1.13,1.41)	1.26(1.14,1.41)	1.25(1.12,1.42)	0.663
NLR	3.13(1.92,5.49)	3.09(1.85,5.59)	3.25(2.08,5.36)	0.512
PLR	98.13(67.82,152.50)	97.87(64.99,151.50)	102.90(72.64,169.70)	0.567
LMR	2.32(1.47,3.29)	2.38(1.51,3.37)	1.97(1.37,3.17)	0.123
**Body composition parameters**				
BMI (kg/m^2^)	23.76 ± 4.11	23.31(20.81,26.04)	24.74(20.88,27.36)	0.062
SMI (cm^2^/m^2^)	44.23(37.42,51.29)	44.03(37.44,51.35)	44.91(37.33,51.20)	0.892
Sarcopenia	130 (27.48)	101 (25.90)	29 (34.94)	0.105
VSR	1.06(0.76,1.46)	0.99(0.71,1.39)	1.30(1.00,1.81)	<0.001
Abnormal adiposity	129 (27.27)	95 (24.36)	34 (40.96)	0.003
VATI (cm^2^/m^2^)	46.48(29.21,65.05)	41.62(25.75,61.81)	59.74(46.69,86.22)	<0.001
High visceral adiposity	301 (63.64)	230 (58.97)	71 (85.54)	<0.001
SATI (cm^2^/m^2^)	40.43(28.8,58.8)	38.38(28.76,57.73)	44.43(28.72,65.97)	0.074
Low subcutaneous adiposity	112 (23.68)	94 (24.10)	18 (21.69)	0.776
TATI (cm^2^/m^2^)	89.81(59.98,123.40)	85.75(56.96,117.60)	109(78.38,154.60)	<0.001
High total adiposity	262 (55.39)	202 (51.79)	60 (72.29)	<0.001

CTP, Child-Turcotte-Pugh classification; MELD, model for end-stage liver disease; MAFLD, metabolic dysfunction-associated fatty liver disease; AILD, autoimmune liver disease; DM, diabetes mellitus; LOH, length of hospitalization; ALT, alanine aminotransferase; AST, aspartate aminotransferase; PT-INR, prothrombin-international normalized ratio; NLR, neutrophil-to-lymphocyte ratio; PLR, platelet-to-lymphocyte ratio; LMR, lymphocyte-to-monocyte ratio; BMI, body mass index; SMI, skeletal muscle index; VSR, visceral to subcutaneous ratio; VATI, visceral adipose tissue index; SATI, subcutaneous adipose tissue index; TATI, total adipose tissue index.

### Distribution of body composition abnormalities

The median IMAC was −0.54 (IQR: −0.63, −0.45) for men and −0.50 (IQR: −0.60, −0.41) for women, and 83 patients (17.55%) were stratified as being myosteatotic. The median SMR was 49.90 (IQR: 43.10, 55.53) HU, with 51.60 (IQR: 45.33, 57.05) HU for male patients and 48.50 (IQR: 40.77, 53.71) HU for female patients. The distribution of myosteatosis was even between male and female participants (19.57 vs. 15.55%, *p* = 0.34). Patients with cirrhosis and myosteatosis had significantly lower levels of SMR in comparison with those without myosteatosis (34.88 vs. 51.65 HU, *p* < 0.001). Surprisingly, there were no significant differences with respect to SMI (44.91 vs. 44.03 cm^2^/m^2^, *p* = 0.892), the presence of sarcopenia (34.94 vs. 25.90%, *p* = 0.105), and a multitude of inflammatory indicators (NLR, PLR, and LMR) between the groups with and without myosteatosis.

The median VSR was 1.34 (IQR: 0.97, 1.81) for men and 0.91 (IQR: 0.65, 1.14) for women, and 27.27% (129/473) of the study population exhibited abnormal adiposity. The median VATI was 50.58 (IQR: 32.74, 66.42) cm^2^/m^2^ for men and 41.41 (IQR: 25.74, 62.50) cm^2^/m^2^ for women, and 63.64% (301/473) of patients showed high visceral adiposity. Finally, the median SATI was 35.99 (IQR: 24.09, 47.17) cm^2^/m^2^ for men and 47.19 (IQR: 31.97, 74.21) cm^2^/m^2^ for women, and 23.68% (112/473) of patients were stratified as low subcutaneous adiposity.

### Determinants of myosteatosis in patients with decompensated cirrhosis

Univariate analyses indicated that advanced age, more aggressive CTP classification, less frequencies of gastroesophageal varices, higher VSR levels, higher SATI levels, higher VATI levels, and higher TATI levels are significantly associated with myosteatosis (Table 3). In multivariate regression model 1, incorporating body composition profiles as continuous variables, the results confirmed that advanced age (*OR* = 1.097, *p* < 0.001), higher VSR (*OR* = 1.574, *p* = 0.032), and higher VATI (*OR* = 1.026, *p* < 0.001) are independent determinants of the presence of myosteatosis in the context of decompensated cirrhosis. In addition, we found that the presence of high visceral adiposity is significantly associated with myosteatosis in model 2 including dichotomized body composition components. For more clarity on the relationship between muscle function and myosteatosis, we have provided additional data by comparing the handgrip strength (HGS) among 149 patients (Supplementary Figure 1). Intriguingly, a trend favoring decreased HGS (16.9 vs. 19.6 kg, *p* = 0.078) was observed in myosteatosis compared with non-myosteatosis of the total population. Notably, male patients with myosteatosis had significantly decreased HGS (22.6 vs. 31.3 kg, *p* = 0.042).

**TABLE 3:** Univariate and multivariate analysis for myosteatosis determined by intramuscular adipose tissue content on CT.

Variable	Univariate analysis			Multivariate analysis					
				Model 1^†^			Model 2^‡^		
	OR	95% CI	*P*	OR	95% CI	*P*	OR	95% CI	*P*
Age (years)	1.091	1.064, 1.119	<0.001	1.097	1.066, 1.128	<0.001	1.097	1.067, 1.127	<0.001
**Sex**									
Male	1.322	0.821, 2.129	0.250						
Female	Reference								
CTP			0.042						
A	Reference								
B	1.584	0.867, 2.894	0.135						
C	2.764	1.251, 6.107	0.012						
GEV	0.525	0.325, 0.848	0.008						
DM	1.541	0.864, 2.749	0.143						
NLR	0.979	0.925, 1.037	0.477						
BMI (kg/m^2^)	1.056	0.997, 1.119	0.065						
Albumin (g/L)	0.964	0.926, 1.004	0.078						
Albumin ≤ 35	1.932	1.005, 3.714	0.048						
SMI (cm^2^/m^2^)	0.991	0.969, 1.013	0.424						
Sarcopenia	1.537	0.927, 2.546	0.095						
VSR	1.949	1.397, 2.720	< 0.001	1.574	1.041, 2.381	0.032			
Abnormal adiposity	2.155	1.314, 3.534	0.002						
SATI (cm^2^/m^2^)	1.009	1.000, 1.018	0.042						
Low subcutaneous adiposity	0.872	0.493, 1.544	0.638						
VATI (cm^2^/m^2^)	1.030	1.020, 1.040	<0.001	1.026	1.015, 1.038	<0.001			
High visceral adiposity	4.116	2.161, 7.838	<0.001				4.084	2.060, 8.097	<0.001
TATI (cm^2^/m^2^)	1.012	1.007, 1.018	<0.001						
High total adiposity	2.428	1.443, 4.084	0.001						

CT, computed tomography; OR, odds ratio; CI, confidence interval; CTP, Child-Turcotte-Pugh classification; GEV, gastroesophageal varices; DM, diabetes mellitus; NLR, neutrophil-to-lymphocyte ratio; BMI, body mass index; SMI, skeletal muscle index; VSR, visceral to subcutaneous ratio; SATI, subcutaneous adipose tissue index; VATI, visceral adipose tissue index; TATI, total adipose tissue index.

^†^Multivariate model 1: age, BMI, CTP, albumin (continuous data), VSR, SATI, VATI, TATI; final model presented.

^‡^Multivariate model 2: age, BMI, CTP, albumin (categorical data), abnormal adiposity, high visceral adiposity, high total adiposity; final model presented.

### Correlation of intramuscular adipose tissue content with age, body mass index, skeletal muscle index, adipose tissue depot indicators, and length of hospitalization

**FIGURE 1:**
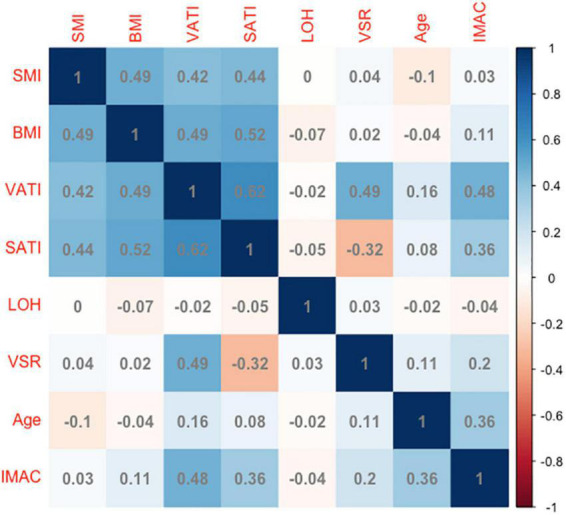
The correlation coefficients of IMAC with age, BMI, SMI, adipose tissue depot indicators, and LOH. IMAC, intramuscular adipose tissue content; BMI, body mass index; SMI, skeletal muscle index; VSR, visceral to subcutaneous ratio of adipose tissue area; VATI, visceral adipose tissue index; SATI, subcutaneous adipose tissue index; LOH, length of hospitalization. Longer LOH > 13 days in the study population.

Next, the basal levels of IMAC were correlated with a multitude of variables to assess their reciprocal relationship. The correlation coefficients of IMAC with age, BMI, SMI, VSR, SATI, VATI, and LOH are demonstrated in Figure 1. The IMAC was positively correlated to the VATI (ρ = 0.48, *p* < 0.001), the SATI (ρ = 0.36, *p* < 0.001), the age (ρ = 0.36, *p* < 0.001), and the BMI (ρ = 0.11, *p* = 0.016), but not significantly correlated with SMI (ρ = 0.03, *p* = 0.690). Moreover, no significant correlation was found between LOH and any body composition components, such as IMAC, VSR, and SMI. In addition, the correlations between basal levels of SMR and a wide array of variables are also shown in Supplementary Figure 2.

### Association between myosteatosis and length of hospitalization

**FIGURE 2:**
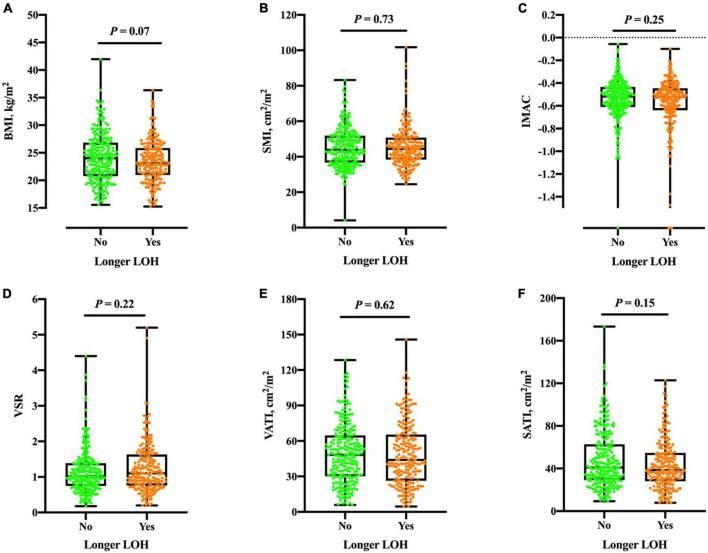
Boxplots of BMI **(A)**, SMI **(B)**, IMAC **(C)**, VSR **(D)**, VATI **(E)**, and SATI **(F)** in different groups according to longer LOH. No significant differences were found between groups of the total population (all *P* > 0.05). BMI, body mass index; SMI, skeletal muscle index; IMAC, intramuscular adipose tissue content; VSR, visceral to subcutaneous ratio of adipose tissue area; VATI, visceral adipose tissue index; SATI, subcutaneous adipose tissue index. Longer LOH > 13 days in study population.

Next, we asked whether myosteatosis may link to one in-hospital outcome pertaining to longer LOH. The median LOH was 13 (IQR: 10, 17) days. In comparison with their counterparts, patients with longer LOH had similar IMAC (and other body composition components) in both genders (Figure 2). Table 4 demonstrates the multivariate logistic regression model to investigate the association of LOH with a spectrum of variables. After fully adjusting for age, gender, and other potential confounders, IMAC was not associated with longer LOH.

**TABLE 4:** Univariate and multivariate analyses for longer LOH among patients with decompensated cirrhosis*.

Variable	Univariate analysis			Multivariate analysis^†^		
	OR	95% CI	*P*	OR	95% CI	*P*
Age (years)	0.993	0.978, 1.008	0.338			
**Sex**						
Male	1.041	0.726, 1.495	0.826			
Female	Reference					
CTP			0.067			
A	Reference					
B	1.643	1.078, 2.504	0.021			
C	1.296	0.682, 2.463	0.429			
MELD	1	0.962, 1.040	0.989			
BMI (kg/m^2^)	0.959	0.917, 1.004	0.072	0.962	0.919, 1.007	0.096
IMAC	0.450	0.181, 1.118	0.085	0.466	0.185, 1.174	0.105
Myosteatosis	0.866	0.537, 1.397	0.556			
SMI (cm^2^/m^2^)	1	0.984, 1.017	0.987			
Sarcopenia	1.336	0.892, 2.003	0.160			
VSR	1.228	0.929, 1.624	0.150	1.273	0.950, 1.705	0.106
Abnormal adiposity	1.487	0.991, 2.233	0.056			
SATI (cm^2^/m^2^)	0.993	0.985, 1	0.058			
Low subcutaneous adiposity	1.159	0.785, 1.772	0.495			
VATI (cm^2^/m^2^)	0.999	0.992, 1.006	0.763			
High visceral adiposity	0.917	0.630, 1.335	0.651			

LOH, length of hospitalization; OR, odds ratio; CI, confidence interval; CTP, Child-Turcotte-Pugh classification; MELD, model for end-stage liver disease; BMI, body mass index; IMAC, intramuscular adipose tissue content; SMI, skeletal muscle index; VSR, visceral to subcutaneous ratio; SATI, subcutaneous adipose tissue index; VATI, visceral adipose tissue index.

*Longer LOH > 13 days in study population.

^†^Multivariate model: age, sex, CTP, BMI, IMAC, SATI, and VSR.

## Discussion

This study provides insights into the potential determinants in relation to the presence of myosteatosis among a multitude of body composition abnormalities, biochemical, hematological, and homeostatic parameters built on the largest samples of decompensated cirrhosis. We identified that advanced age, higher VSR, and higher VATI are independently associated with myosteatosis. These findings may embrace clinical implications with regard to interventions reducing visceral adipose tissue accumulation, along with fostering muscle quality as therapeutic targets among cirrhotics. Furthermore, the muscle quality indicator estimated by IMAC was unable to predict longer LOH, in line with previous findings. That is, the prognostic utility of myosteatosis appears to be particularly significant in the long-term outcomes (3, 7).

There has been a surge in the evaluation of various body composition and their clinical implication in relation to inferior outcomes among cirrhotics (15). Unlikely sarcopenia, the best-recognized body composition abnormality affecting both morbidity and mortality, investigation of the prognostic role, clinical utility, predisposing factor, and pathobiological mechanisms of myosteatosis is still elusive. We and others have demonstrated that myosteatosis is linked to mortality, the presence of minimal hepatic encephalopathy, the development of overt hepatic encephalopathy, and frail phenotype among cirrhotics (4, 5, 10). However, limited research has explored the risk factor or illustrative issue in relation to the presence of myosteatosis, while relevant reports are even sparse in the context of cirrhosis. Notably, Baker et al. showed that intramuscular fat accumulation and resultant low muscle density are significantly associated with chronic inflammation, smoking, low muscle mass, and abundant visceral adiposity in patients with rheumatoid arthritis (16). Another report indicated that female gender, older age, concomitant sarcopenia, and higher VSR are factors independently associated with myosteatosis among 362 subjects with chronic liver disease (8). Our results extend the knowledge of a previous report evaluating the risk factors of myosteatosis in the setting of patients with cirrhosis by highlighting increased VATI as a predisposing factor rather than wasting of skeletal muscle volume. We suppose that liver disease severity (compensation vs. decompensation), cutoff applied for identifying sarcopenia (on the basis of population norms vs. patient mortality), and approach used for measuring myosteatosis (muscle attenuation vs. IMAC) may account for these disparities.

Our results are to an extent discordant with other studies, and therefore merit an in-depth statement. Actually, it was not unexpected that the higher VATI and TATI serve as predisposing factors to the advent of myosteatosis. At anatomical levels, two types of fat accumulation within the skeletal muscle have been identified, that is, inter-muscular adipose tissue (fat beneath deep fascia and between corresponding muscle groups) and intra-muscular adipose tissue (fat within muscle fibers). The former is represented by the density/volume of the adipose tissue, while the latter is characterized by the muscle density (higher IMAC indicates greater fat depot in the muscle tissue) (17). Moreover, intramuscular fat can negatively impact muscle quality by distorting muscle fibers’ alignment, and as a consequence, weakening mechanical action (18). Overwhelming lipid availability may flux into other locations, resulting in ectopic lipid deposition in the skeletal muscle and lipotoxic intermediated accumulation (19). Furthermore, inadequate storage of lipids in the subcutaneous adipose tissue is responsible for abnormal fat storage in VAT as well as muscle (i.e., myosteatosis) (20, 21). Greater fat expansion in visceral depots and increased VATI may give rise to chronic inflammation and insulin resistance (22, 23). On the other hand, mounting evidence has highlighted the notion that the distribution of adipose tissue rather than the absolute volume of its respective depot is closely associated with disease severity. For instance, Ha et al. clearly showed that increased VSR measured on CT serves as an imaging biomarker related to histologic VAT inflammation manifested by lymphoplasmacytic aggregates (24). Taken together, we believe that the clinical relevance of VATI/VSR and their relationship to myosteatosis reflect multiple facets, such as ongoing inflammatory activity, hormonal homeostasis dysregulation, and disease progression.

Another critical issue to identify is the illustrative issue pertaining to myosteatosis accounts for developing appropriate therapeutic strategies. Actually, some pioneers addressed an improvement in various body composition profiles following interventional treatment. Gioia and colleagues found that sarcopenia and myosteatosis greatly improved in response to transjugular intrahepatic portosystemic shunt (TIPS) placement (25). Intriguingly, SATI significantly increased in addition to the drastic decrement pertaining to VATI, which was relevant to the amelioration of hepatic encephalopathy episodes independent of hepatic function. Similarly, another study in China observed that skeletal muscle (SMI) and fat mass (subcutaneous fat area) persistently increase at 5 months following TIPS and remain stable at 1-year follow-up in sarcopenic cirrhotics (26). Collectively, it should be noted that interventions targeting different adipose tissue depots alongside maintaining or gaining muscle quantity appear to be practical in the context of cirrhosis. Given the potential contribution of VATI/VSR uncovered in the present study, we hold the promise that TIPS may effectively restore adipose tissue volume or ratio to normal levels, aiming at the prevention of myosteatosis in a specific subgroup of choice.

After careful retrieval from the PubMed database, we located scattered studies exploring the predictive utility of myosteatosis for longer LOH in patients with cirrhosis. The impact of myosteatosis on LOH remains a matter of debate. A study recruiting 180 patients undergoing transplantation demonstrated perioperative myosteatosis, estimated by MRI in terms of the fat fraction of erector spinae muscles, is associated with increased LOH (27). Another report on 106 liver transplantation recipients implicated that patients with myosteatosis, who were diagnosed according to the L3 muscle attenuation radiodensity, spent an average of 6 days longer in the hospital (28). In contrast, no difference in the LOH was observed in a large cohort of 678 patients with cirrhosis between groups, despite the same measurement methods and levels having been applied as in the study mentioned above (3). The current study described, using our prerequisite cutoffs for patient mortality on CT, revealed no relationship between myosteatosis and LOH. These discrepancies can be attributed to different anatomical landmark, imaging modality for measurement, and heterogeneous population of study for analysis.

Our study has several limitations. First, casualty between risk factors and the development of myosteatosis cannot be made due to the retrospective nature of the study. It is more likely for practitioners to delineate an illustrative issue pertaining to myosteatotic phenotype after reading this article. However, our preliminary results are crucial for instigating further multicenter studies linking myosteatosis to varying inferior health outcomes, such as frailty assessed by different approaches (Liver Frailty Index, etc.) (29). Second, there is currently a lack of standard modalities and definitions of myosteatosis in the context of cirrhosis, which impedes the generalizability of our findings. Third, the prevalence of myosteatosis in the current study is dramatically lower than that of other investigations. Although the reasons remain elusive, we speculate that racial characteristics, dietary regimens, body habitus, and lifestyles between Asian and Western individuals may account for these disparities.

In conclusion, higher VSR, higher VATI, and advanced age are associated with myosteatosis among patients with cirrhosis at the decompensation phase. It is tempting to target divergent adipose tissue depots aimed at timely intervention/prevention of myosteatosis.

## Data availability statement

The raw data supporting the conclusions of this article will be made available by the authors, without undue reservation.

## Ethics statement

The studies involving human participants were reviewed and approved by Ethics Committee of Tianjin Medical University General Hospital. The patients/participants provided their written informed consent to participate in this study.

## Author contributions

XW, MS, YL, and CS designed the study, analyzed the data, and prepared the original draft. LM, GG, and WY conducted the study and edited the manuscript. YH and ZY analyzed the data and reviewed the manuscript. XF and BC collected the data and conducted the statistical analysis. KJ and CS designed and monitored the study and made critical revisions to the manuscript. All authors contributed to the article and approved the submitted version.

## Footnotes

1
http://www.r-project.org/
