# Supplementary material for: Association of myosteatosis with various body composition abnormalities and longer length of hospitalization in patients with decompensated cirrhosis
Source: Front Nutr. 2022 Sep 15;9:921181. doi: 10.3389/fnut.2022.921181 (PMC9520990; doi:10.3389/fnut.2022.921181)
Supplement: Supplementary file 1 [file Presentation_1.pdf]

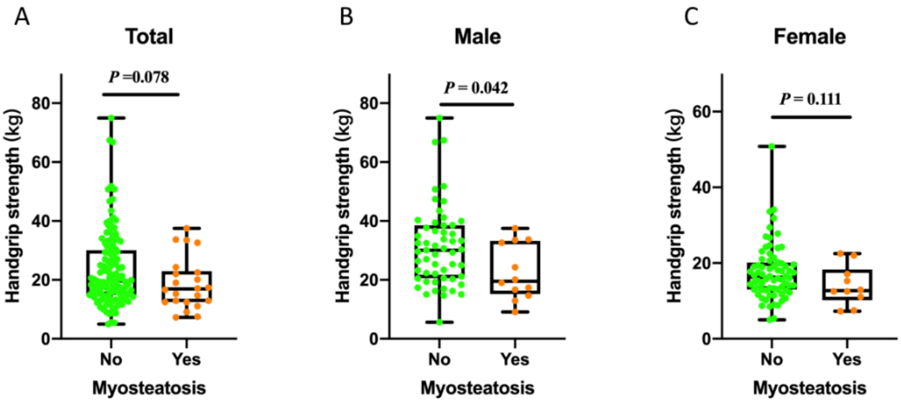

Figure S1 Boxplots of handgrip strength in different groups according to the presence of myosteatosis of the total population (A), male patients (B) and female patients (C) with cirrhosis. The handgrip strength was evaluated using a dynamometer (EH101; CAMRY, Guangdong, China). Participant were instructed to stand upright, and the dynamometer beside should not be against their bodies. They were asked to experience maximum force trial for the dominant hand, and the results were recorded in kg. The best performance of three efforts was indicative of the final score.

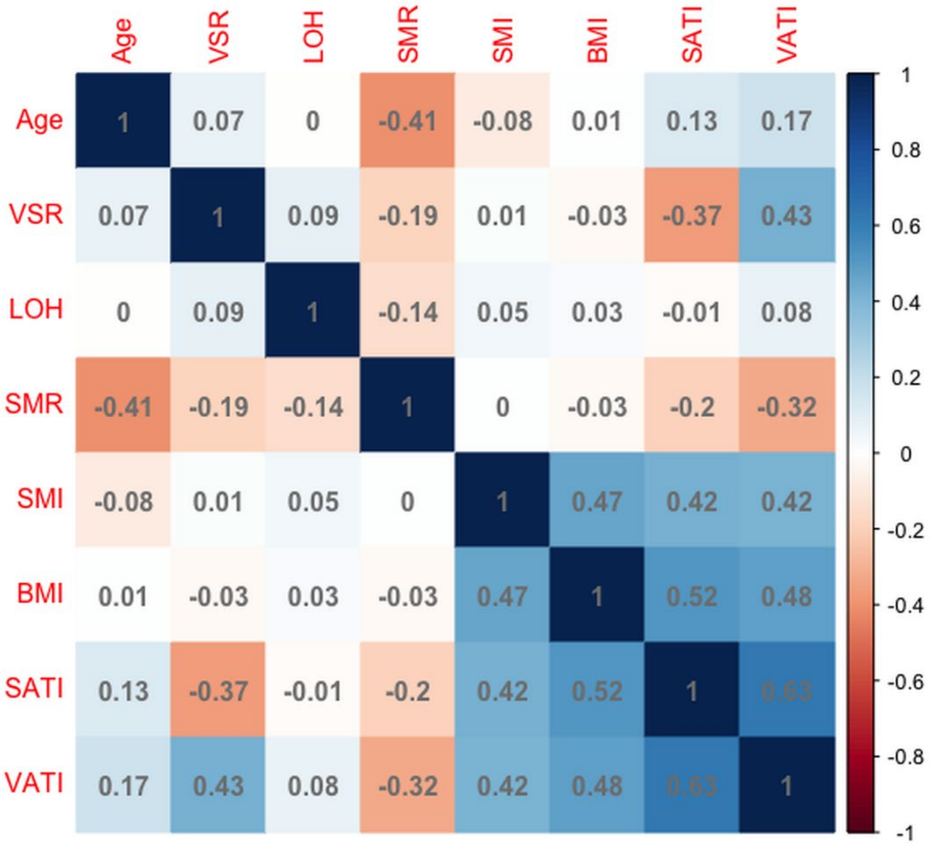

Figure S2 The correlation coefficients of SMR with age, BMI, SMI, adipose tissue depot indicators and LOH. SMR, skeletal muscle radiodensity; BMI, body mass index; SMI, skeletal muscle index; VSR, visceral to subcutaneous ratio of adipose tissue area; VATI, visceral adipose tissue index; SATI, subcutaneous adipose tissue index; LOH, length of hospitalization. Longer LOH >13 days in study population. The SMR was negatively correlated to the VATI (ρ = -0.32, *P* <0.001), the SATI (ρ = -0.20, *P* <0.001), the age (ρ = -0.41, *P* <0.001) and the VSR (ρ = -0.19, *P* <0.001).
